# Methodological literature on the reporting of systematic reviews of health economic evaluations: a scoping review protocol

**DOI:** 10.12688/f1000research.156907.1

**Published:** 2024-11-18

**Authors:** Phuong Bich Tran, Joseph Kwon, Anastasios Bastounis, Stavros Petrou, Andrew Booth, PRISMA-EconEval Management Group

**Affiliations:** 1 Nuffield Department of Primary Care Health Sciences, University of Oxford, Oxford, UK; 2 Sheffield Centre for Health and Related Research (SCHARR), University of Sheffield, Sheffield, UK

**Keywords:** reporting, systematic review, health economic evaluation, review protocol

## Abstract

Systematic reviews of health economic evaluations play a crucial role in informing evidence-based healthcare decisions, yet they lack standardized reporting guidelines. A project has been initiated that aims to extend the Preferred Reporting Items for Systematic Reviews and Meta-Analyses (PRISMA) guideline for systematic reviews of health economic evaluations (PRISMA-EconEval). This scoping review forms a foundation for the PRISMA-EconEval project, aiming to identify, map, and extract candidate reporting items from the methodological literature.

The scoping review will follow the PRISMA Extension for Scoping Reviews (PRISMA-ScR) Checklist and involve comprehensive searches in databases such as PubMed MEDLINE, Embase, and Web of Science, covering the period from 2015 to 2024. Supplementary searching, reference checking and citation searching will target grey literature, overlooked studies and evidence prior to 2015. Inclusion criteria will focus on methodological papers that provide frameworks or recommendations for reporting systematic reviews of health economic evaluations and enhanced case studies that critically discuss methods and reporting structures. The extracted data will be coded and analyzed to produce an initial list of candidate reporting items, structured according to conventional sections of a systematic review (e.g., title, abstract, methods, results). This initial list will be used in the subsequent stages of the project and disseminated through a peer-reviewed publication and presentations at international conferences.

The outcome of this scoping review will significantly contribute to the development of a comprehensive PRISMA-EconEval reporting guideline, aimed at enhancing the transparency, consistency, and quality of systematic reviews of health economic evaluations, and provide an essential tool for authors, editors, peer-reviewers, and stakeholders.

## Introduction

A systematic review summarizes the available evidence and provides an unbiased, reliable assessment of the current state of knowledge, essential for making evidence-based decisions. ^1^ The Preferred Reporting Items for Systematic reviews and Meta-Analyses (PRISMA) 2020 statement was designed to enhance the reporting of systematic reviews and meta-analyses – primarily of the effects of interventions, though also applicable to systematic reviews and meta-analyses of studies with a different objective – by improving their clarity, consistency, transparency, quality, and overall value. ^2^

Systematic reviews of health economic evaluations differ from other systematic reviews in their approach to reporting key methodological steps, including methods for study selection, data extraction and presentation, and the assessment of quality and relevance. Such reviews consider health economic evaluations, which compare the effects of different healthcare interventions on economic outcomes (e.g. incremental cost-effectiveness ratios, net monetary benefits, etc.). Additionally, they differ in their potential for combining costs, preference-based health-related quality of life outcomes, and cost-effectiveness outcomes across studies.

A literature search in PubMed Central from January 1, 2015 to March 25, 2017 found 202 systematic reviews of health economic evaluations listed within this 27-month period. ^3^ Extending the search to other databases and grey literature and from 2017 to date is likely to increase this number substantially. These reviews lack common standards for reporting quality, highlighting the need for an additional reporting guideline specifically for systematic reviews of health economic evaluations.

The ‘Development of a PRISMA extension for systematic reviews of health economic evaluations (PRISMA-EconEval)’ project was initiated to address this gap. A core international multidisciplinary working group, including PRISMA members, authors, journal editors, and stakeholders, will identify and extract candidate reporting items from various sources. Such sources include this scoping review of the methodological literature on the reporting of systematic reviews of health economic evaluations together with a purposive sample of previous systematic reviews of health economic evaluations. They will refine these items to improve reporting quality. A multi-round online Delphi survey will then evaluate these items with input from academic researchers, specifically health economists (including authors of systematic reviews of health economic evaluations) and systematic review methodologists, journal editors, reporting guideline developers, information specialists, and stakeholders, including health care decision-makers, health research funders, and patient and public representatives. The development of common reference standards for reporting should enhance completeness, transparency and structure in the reporting of systematic reviews of health economic evaluations and generate user-friendly tools for authors, editors, peer-reviewers and stakeholders that facilitate reporting.

As a first step, it is necessary to review the methodological literature on the reporting of systematic reviews of health economic evaluations. This process will help map the current methods, guidance, and recommendations for reporting such reviews and is part of the process of identifying common reference standards for reporting them.

Research objectives

1. To identify and review the methodological literature on the reporting of systematic reviews of health economic evaluations.
2. To summarize and map methods, guidance, and recommendations for reporting such reviews.
3. To identify and extract an initial dataset of candidate reporting items.

## Method

### Protocol

The scoping review forms part of the larger PRISMA-EconEval project. The protocol for the scoping review follows the Preferred Reporting Items for Systematic Review and Meta -Analysis Protocols (PRISMA-P) 2015 Checklist, ^4^ with the protocol and PRISMA-P checklist deposited on the Open Science Framework. ^5^ We will report the scoping review in accordance with the Preferred Reporting Items for Systematic reviews and Meta-Analyses extension for Scoping Reviews (PRISMA-ScR) Checklist. ^6^

**Figure 1.**
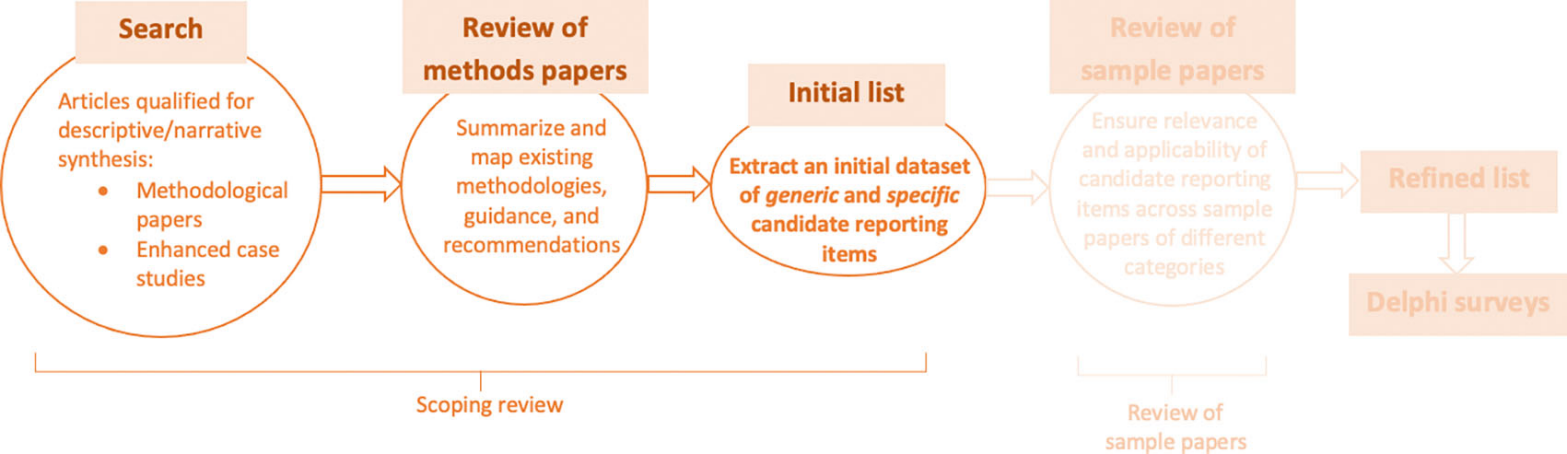
Stages of the PRISMA-EconEval project.

The scoping review will be conducted in various stages as shown in Figure 1 and will aim to identify an initial list of candidate reporting items. This initial list of reporting items will have generic relevance to systematic reviews of health economic evaluations regardless of the types of health economic evaluation (e.g., cost-minimization analysis, cost-benefit analysis, cost-effectiveness analysis, cost-utility analysis, cost-consequences analysis) and the vehicles for health economic evaluation (e.g., economic evaluations based on randomized controlled trials, economic evaluations based on observational studies with patient-level data, decision-analytic models) adopted by the individual studies they cover. Specific reporting items relevant to different categories of health economic evaluations may also be identified. The initial list will then be enhanced by findings from an additional sample of published systematic reviews of health economic evaluations. After refinement by the PRISMA-EconEval Management Group, this list will inform the multi-round online Delphi survey. Detailed plans for subsequent stages (post-scoping review) will be outlined in the comprehensive PRISMA-EconEval project protocol.

The scoping review will focus on two types of articles (either published in journals or elsewhere):

• Methodological papers: Articles that describe methods, frameworks, guidance for conduct, or recommendations on the reporting of systematic reviews of health economic evaluations.
• Enhanced case studies: Systematic reviews of health economic evaluations that include a minimum of two paragraphs that discuss methods, conceptual approaches, limitations (e.g., items not analyzed/reported in the review), and/or thorough categorization and reporting of health economic evaluation features that can inform the initial list of candidate reporting items.

### Conditions or domains being studied

We used the ECLIPSE Framework to help in framing the scope and objectives of the scoping review ( Table 1). ^7^

**Table 1.** The ECLIPSE framework.

Element	Definition	Description
**E**xpectation	What are you looking to improve or change? What is the information going to be used for?	We aim to collate and summarize methods, guidance, and recommendations for the reporting of systematic reviews of health economic evaluations. This is an initial step in the development of a new reporting guideline for systematic reviews of health economic evaluations.
**C**lient group	Who is the service or policy aimed at?	Authors, editors, peer-reviewers and stakeholders that conduct or facilitate the reporting of systematic reviews of health economic evaluations.
**L**ocation	Where is the service or policy located?	Unrestricted
**I**mpact	What is the change in service or policy that the researcher is investigating?	Findings from the scoping review will form the initial dataset of candidate reporting items, which is a key step in developing the PRISMA-EconEval guideline. The development of a common reference standard for reporting should enhance completeness, transparency and structure in the reporting of systematic reviews of health economic evaluations.
**P**rofessionals	Who is involved in providing or improving the service or policy?	PRISMA-EconEval Management Group, Independent Advisory Group, Delphi Panelists, Patient and Public Involvement Group, Pilot End-Users
**Se**rvice	What kind of service or policy is this?	The development of a reporting guideline

### Expected timeline for scoping review

August 2024 – December 2024.

### Search strategy

We will conduct searches in PubMed MEDLINE, Embase, and Web of Science for the period from 2015 to 2024. Relevant papers published prior to this timeframe will be identified through supplementary searching, reference checking and citation searching.

For the grey literature, we will identify and agree upon a list of 10-12 websites of health technology assessment agencies or health economics associations that may publish methods papers, guidance, and recommendations for reporting systematic reviews of health economic evaluations.

### Search terms and strings

An experienced information specialist (ABo) will search databases of the published literature, applying guidance on filters at the intersection of “systematic reviews” and “economic evaluations” to develop and pilot our search strategies, including data sources, date limits, search terms, and syntax ( Box 1).

Box 1. **Search string examples for PubMed.**

“systematic review of economic evaluations”[tiab:~0] OR “systematic review of cost effectiveness”[tiab:~0] OR “systematic review”[PT] AND (economic*[Ti] OR (“economic evaluation*” OR cost [Ti] OR costs [Ti] OR “cost effectiveness” OR “cost benefit analysis” OR “Cost Utility Analysis” OR “Cost-Benefit Analysis”)

“systematic reviews of economic evaluations”[tiab:~0] OR “systematic reviews of cost effectiveness”[tiab:~0] OR ((“Systematic Reviews as Topic” OR “Review Literature As Topic”) AND (economics [SH] OR economic*[Ti] OR cost [Ti] OR costs [Ti] OR “cost effectiveness” OR “cost benefit analysis” OR “Cost utility Analysis” OR “Cost-Benefit Analysis”)) OR (“systematic reviews” AND (“economic evaluation*” OR cost [Ti] OR costs [Ti] OR “cost effectiveness” OR “cost benefit analysis” OR “Cost utility Analysis” OR “Cost-Benefit Analysis”) AND (Methods OR methodology OR methodological OR checklist* OR guideline*)) OR ((“Cost-Benefit Analysis” AND (Methods OR methodology OR methodological OR checklist* OR guideline*) AND (“Systematic Reviews as Topic” OR “Review Literature As Topic”))

For the grey literature, the scoping review team will search the websites of health technology assessment agencies and health economics associations, using similar search terms.

### Supplementary search methods

Recognizing that methodological considerations are not always adequately reported in the titles and abstracts of systematic reviews of health economic evaluations, the research team will use innovative supplementary search methods to access the contents of full text articles and establish their eligibility for inclusion. Scite (open access alternative — Consensus) and Litsense will be interrogated for “systematic reviews of (health) economic evaluations”. Google Scholar will be searched using targeted search terms via the free open source Publish or Perish search tool to access full text contents of high currency and specificity. We will also use BASE, ^8^ one of the largest search engines for academic web resources, which captures both academic and grey literature that may have been missed. Finally, a list of 10-12 known “index” items of high relevance to the review question will be used as the starting point for both backwards citation chaining (reference checking) and forwards citation chaining (citation chasing) using the Citation Chaser open-source tool.

### Inclusion and exclusion criteria

For methodological papers, we will include articles that describe methods, frameworks, guidance, or recommendations on the reporting of systematic reviews of health economic evaluations. For enhanced case studies, we will include systematic reviews of health economic evaluations that contain at least two paragraphs critically discussing methods, conceptual approaches, limitations (e.g., items that were not reported but should have been), and/or thorough categorization and reporting of health economic evaluation features that can inform the initial list of candidate reporting items. Both types of articles published in English will be included without date restriction. We will exclude conference abstracts and proceedings, except for those that specifically focus on reporting methodologies of systematic reviews of health economic evaluations. For these, we will conduct additional searches and contact the authors (if necessary) to determine if a full-text publication is available. If a full-text version cannot be identified, they will be excluded.

### Article screening and selection

References for the articles retrieved will be uploaded to Covidence ^9^ (open access alternative — Rayyan) for deduplication and initial screening (Phase One) and, subsequently, for full-text screening and reference management (Phase Two).

The eligibility of each identified study will be assessed by two of the five reviewers (ABa, ABo, JK, PT, SP) independently. A two-stage standard protocol will be developed and followed via checklists for each of the two phases. The checklists will be piloted on 100 random articles and screening criteria adapted accordingly.

*Phase One screening (Title and abstract screening):* The following checklist will be used for the first screening of articles after deduplication ( Table 2). All articles will be classified accordingly. Each independently classified article by every two reviewers will be automatically compared by Covidence. Disagreements between reviewers will be resolved through consensus or by referral to a third reviewer.

**Table 2.** Phase One screening checklist.

Q	Question	Answer	Action following answer	Comments/examples
**1**	**Based on title/abstract, is the article likely to focus on reporting methodologies for systematic reviews/evidence syntheses of health economic evaluations?**	Yes	Classify as **“Retained phase 1, methodological paper**”	The primary focus of the article should be on methods, frameworks, guidance, or recommendations on the reporting of systematic reviews of health economic evaluations.
		No	Go to Q2	
		Maybe	Go to Q2	
**2**	**Based on title/abstract, is the article a systematic review of health economic evaluations: i.e., a ‘worked example’? Alternatively, is it likely to focus on such reviews, or at minimum, on health economic evaluations?**	Yes	Go to Q3	The article should at minimum, focus on full health economic evaluations or systematic reviews of such studies. Review of reviews may also be helpful. Partial health economic evaluations (e.g., cost analyses, utility elicitation studies) should be excluded.
		No	Classify as “Exclude”	
		Maybe	Go to Q3	
**3**	**Does the title/abstract discuss issues relevant to or contain useful information regarding the reporting of systematic reviews of health economic evaluations?**	Yes	Classify as **“Retained phase 1, enhanced case study”**	In the case of ‘worked examples’, the abstract may show hints of discussions related to the reporting of systematic reviews of health economic evaluations. (e.g., *Studies demonstrated variation or heterogeneity in reporting; XX studies did not include the perspective of the analysis, and YY studies failed to specify the choice of discount rate.*) The abstract may also include thorough categorizations that could inform the list of candidate reporting items. (e.g., *Cost-effectiveness was estimated using partitioned survival models in XX studies and state-transition models in YY studies. Across studies, XX reported utility values by health state and YY reported disease-specific HRQoL.*)
		No	Classify as “Exclude”	
		Maybe	Classify as **“Retained phase 1, potential enhanced case study”**	

*Phase Two screening (Full-text screening):* For all articles classified as “Retained Phase 1”, the full article will be read and further classified as below ( Table 3). Disagreements between reviewers will be resolved through consensus or by referral to a third reviewer.

**Table 3.** Phase Two screening checklist.

Q	Question	Answer	Action following answer	Comments/examples
**1**	**Does the article focus on reporting methodologies for systematic reviews/evidence syntheses of health economic evaluations?**	Yes	Classify as **“Include, methodological paper**”	The primary focus of the article should be on methods, frameworks, guidance, or recommendations on the reporting of systematic reviews of health economic evaluations.
		No	Go to Q2	
		No	Classify as “Exclude”	
**2**	**Does the article contain at least two paragraphs critically discussing methods, conceptual approaches, limitations and/or recommendations for the structuring and reporting of systematic reviews of health economic evaluations. Alternatively, does it contain useful information regarding the reporting of systematic reviews of health economic evaluations?**	Yes	Classify as **“Include, enhanced case study”**	In the case of ‘worked examples’, the article may (e.g., in the discussion section) discuss issues relating to the reporting of systematic reviews of health economic evaluations. In the result section, they may also discuss items that were not reported but should have been. (e.g., *Studies demonstrated variation or heterogeneity in reporting; XX studies did not include the perspective of the analysis, and YY studies failed to specify the choice of discount rate.*) The article may also include thorough categorizations that could inform the list of candidate reporting items. (e.g., *Cost-effectiveness was estimated using partitioned survival models in XX studies and state-transition models in YY studies. Across studies, XX reported utility values by health state and YY reported disease-specific HRQoL.*)
		No	Classify as “Exclude”	

### Data extraction and coding

Data from studies that fulfill the inclusion criteria will be entered (by ABa, ABo, JK, PT, SP) onto an Excel form. Data to be extracted will include: article PMID number, title of paper, authors, year of publication, journal or source, type of literature (i.e., published scientific article, grey literature), and type of paper (i.e., methodological paper, enhanced case study). Recommendations from included studies on the methods and reporting of systematic reviews of health economic evaluations will be extracted and organized by codes and sub-codes. The coding will be conducted using NVivo V.1.7 ^10^ (open access alternative — QualCoder). The reviewers will work together using a shared open codebook. All domains described in the included studies will be gathered and tabulated. Conventional sections and sub-sections of a systematic review as informed by the PRISMA guideline (i.e., title, abstract, introduction, methods, results, discussion, other information) will be used to inform the coding by which the identified themes and topics are organized. The extraction process will follow a pilot round of coding, continual refinement and revision of the codebook, and frequent consensus meetings.

### Data analysis and reporting

The objective of the scoping review is to produce an initial list of recommended reporting items for systematic reviews of health economic evaluations, to be used in subsequent stages of the PRISMA-EconEval project. Where necessary, items will be renamed/rephrased, similar items will be combined, and items will be split if they cover two or more distinct topics.

The initial list of candidate reporting items identified by the PRISMA-EconEval Management Group will be arranged according to the conventional sections of a systematic review: title, abstract, introduction, methods, results, discussion, and other information. This structure broadly aligns with other extensions to the PRISMA reporting guidelines. The initial list of candidate reporting items will have *generic* relevance to systematic reviews regardless of the types of health economic evaluation or the vehicles for health economic evaluation adopted by the individual studies they cover. We will also include *specific* reporting items relevant to different categories of health economic evaluations in this and subsequent stages of the PRISMA-EconEval project.

### Dissemination

We plan to disseminate the findings of the scoping review by submitting the resulting manuscript to a suitable peer-reviewed journal and presenting the key findings at the 2025 International Health Economics Association Congress. The initial list of candidate reporting items will be used in the subsequent stages of the project; i.e., review of sample papers and the Delphi surveys.

### Study status

The protocol has been registered on the EQUATOR Network database ^11^ and the Open Science Framework. ^5^ The database searches have been conducted and pilot screening will soon take place.

### Ethical considerations

Ethical approval is not required for this aspect of the PRISMA-EconEval project as it does not involve individual or personal data.

## Author contributions

The review was conceptualized collectively by the team (PT, JK, ABa, SP, ABo). PT developed the initial draft manuscript and created visualizations, with reviews and edits by the team. ABo and ABa provided expertise on the review methodology, databases and search strategy. PT was responsible for the revision and submission of the manuscript and is the guarantor of the protocol. SP, JK, ABo provided supervision throughout the entire process. All authors have reviewed and approved the final version of the manuscript.

List of members of the PRISMA-EconEval Management Group

1. Prof Stavros Petrou, Nuffield Department of Primary Care Health Sciences, University of Oxford, United Kingdom
2. Dr Joseph Kwon, Nuffield Department of Primary Care Health Sciences, University of Oxford, United Kingdom
3. Dr Phuong Bich Tran, Nuffield Department of Primary Care Health Sciences, University of Oxford, United Kingdom
4. Prof Andrew Booth, Sheffield Centre for Health and Related Research (SCHARR), University of Sheffield, United Kingdom
5. Dr Anastasios Bastounis, Sheffield Centre for Health and Related Research (SCHARR), University of Sheffield, United Kingdom
6. Prof Sophie Staniszewska, RCN Research Institute, Warwick Medical School, University of Warwick, United Kingdom
7. Mr Richard Grant, Patient and Public Involvement Representative, United Kingdom
8. Prof Sally Hopewell, Nuffield Department of Orthopaedics, Rheumatology and Musculoskeletal Sciences, University of Oxford, United Kingdom
9. Dr Matthew Page, Methods in Evidence Synthesis Unit, School of Public Health and Preventive Medicine, Monash University, Australia

## Data Availability

No underlying data is associated with this article. Reporting guideline. Open Science Framework. [Development of new reporting guidance for systematic reviews of health economic evaluations (PRISMA-EconEval)] DOI: https://doi.org/10.17605/OSF.IO/FSPG9. ^5^ This project contains the following data:
−
PRISMA-EconEval Scoping Review Protocol.pdf
−
PRISMA-P-checklist.pdf Data are available under the terms of the Creative Commons Attribution 4.0 International license (CC-BY 4.0).
