# Supplementary material for: Methodological literature on the reporting of systematic reviews of health economic evaluations: a scoping review protocol
Source: F1000Res. 2024 Nov 18;13:1382. [Version 1] doi: 10.12688/f1000research.156907.1 (PMC11865710; doi:10.12688/f1000research.156907.1)
Supplement: Peer review history

# Reviewer report: Reviewer response for version 1

**DOI:** 10.5256/f1000research.172286.r363770

**Published:** 2025-02-26

**Authors:** Marta Trapero-Bertran

**Affiliations:** 1 Department of Economics and Business, Biomedical Research Institute of Lleida, University of Lleida, Advisory Committee on the Financing of Pharmaceutical (CAPF), Lleida, Spain

I would like to congratulate you on this outstanding piece of work. Your article is highly engaging, well-written, and clearly articulated, making it a valuable contribution to the academic field.

 The only comment I would like to make relates to the search terms used in your bibliographic review. Rather than concatenating all the suggested search terms as an example of a search strategy in Medline, it might be more efficient to use the * symbol to avoid explicitly listing all possible variations of economic evaluation types. In this regard, I would suggest something like the following search strategy:

"systemat* rev*" AND ("cost*" OR "econ* eval*") AND ("checklist*" OR "method" OR "anal*")**

 This adjustment could help broaden the search results while maintaining specificity, and shortening search terms used.

 Once again, congratulations on this excellent article, and thank you for your valuable contribution.

Is the study design appropriate for the research question?

Partly

Is the rationale for, and objectives of, the study clearly described?

Yes

Are sufficient details of the methods provided to allow replication by others?

Yes

Are the datasets clearly presented in a useable and accessible format?

Not applicable

Reviewer Expertise:

Health Economics and Economic Evaluation

I confirm that I have read this submission and believe that I have an appropriate level of expertise to confirm that it is of an acceptable scientific standard.

# Reviewer report: Reviewer response for version 1

**DOI:** 10.5256/f1000research.172286.r363774

**Published:** 2025-02-20

**Authors:** Vicki Brown

**Affiliations:** 1 Deakin Health Economics, Institute for Health Transformation, Deakin University, Geelong, Australia

This paper presents the protocol for a scoping review of systematic reviews of health economic evaluations, with the aim of informing the development of a PRISMA "add-on" for reporting of SRs of health economic evaluations.  The manuscript is clear and well written.  The methods proposed are sound.  I have no comments or edits.

Is the study design appropriate for the research question?

Yes

Is the rationale for, and objectives of, the study clearly described?

Yes

Are sufficient details of the methods provided to allow replication by others?

Yes

Are the datasets clearly presented in a useable and accessible format?

Not applicable

Reviewer Expertise:

Health economics

I confirm that I have read this submission and believe that I have an appropriate level of expertise to confirm that it is of an acceptable scientific standard.

# Reviewer report: Reviewer response for version 1

**DOI:** 10.5256/f1000research.172286.r347340

**Published:** 2025-01-07

**Authors:** Ghislaine A.P.G. van Mastrigt

**Affiliations:** 1 Department of Health Services Research, Care and Public Health Research Institute (CAPHRI), Maastricht University, Maastricht, The Netherlands

This study aims to investigate the existing literature through a scoping review to develop an alternative to the well-established PRISMA reporting guidelines, namely one to improve the reporting quality of health economic evaluations. Overall, this is a very well-written and clear protocol for performing this research. However, the authors were given a few suggestions to improve this scoping review protocol.

 Regarding the questions of the review report form:

Is the rationale for, and objectives of, the study clearly described?

 Partly

 The authors report the three research objectives:

- 1. To identify and review the methodological literature on reporting systematic reviews of health economic evaluations.
- 2. To summarize and map methods, guidance, and recommendations for reporting such reviews.
- 3. To identify and extract an initial dataset of candidate reporting items.

For objective 1, I would not solely focus on ‘reporting’ whether you can identify all the relevant literature; I would rephrase it as follows: To identify and review the methodological literature of systematic reviews of health economic evaluations to improve the reporting quality of these studies.

 For objective 2: it would rephrase it as follows: To summarize and map methods, guidance, and recommendations for methodological literature of systematic reviews of health economic evaluations.

 For objective 3: to identify and extract an initial dataset of candidate items to develop an extension to PRISMA guidelines for Health economic evaluations

 In summary, what aspects do you consider as part of reporting economic evaluations?  This could be specified for the objectives, but this is also needed for the protocol.

Is the study design appropriate for the research question?

 Yes

 The authors propose a scoping review to investigate the existing literature on this topic, I agree this is an appropriate research design to investigate this research question.

Are sufficient details of the methods provided to allow replication by others?

 Partly

 Regarding the step of the selection of the databases: They state: ‘’ We will conduct searches in PubMed MEDLINE, Embase, and Web of Science for the period from 2015 to 2024’’. However, no rationale behind the choices of these bibliographic databases is given; for economic topics; Econlit could also be a relevant database to check.

**Other topics**

**General topic: study designs**

 For the scoping review but also the PRISMA-EE development; what kind of types of economic evidence do the authors want to cover in their reporting guidelines? only full economic evaluations: like cost-utility or cost-effectiveness analyses or also partial economic evaluations like; cost of illness studies, resource use etc. This is explained in the phase one checklist but should also be more clearly mentioned in the protocol.

 It is highly recommended to start the study after the registration of the protocol; so please adapt the start date of review to January 2025.

**Sections: search strategy/search terms and strings**

 This section could be improved by adding additional information on search strategy development and databases selected (for details see comments below).

 It is highly recommended to report the search strings for each of the bibliographic databases which will be used to identify relevant studies. In addition, it is also highly recommended to use validated and predefined search strings as much as possible. The ISSG website is a recommended source to look for, especially for the economic topics (table needs further specification). Furthermore, more details could be given on how the search strategies were developed. For this the Prisma extension ‘’ PRESS’’ is very helpful.

 The search strategies listed in box 1 do not match the study objectives and could be made more specific.

 Details of the 10-12 websites of health technology assessment agencies or health economics associations could be added in an appendix.

 Please also specify the 10-12 ‘’index items’’ mentioned to be used as starting points for backward and forward citation chasing. ‘’Citation chasing’’ is mentioned twice, once briefly at the beginning of the section and more detail at the end, one of them could be deleted.

**Section: Inclusion and exclusion criteria**

 Details regarding the type of economic evaluation included, approaches used (e.g. model/trial-based), as well as including full or also partial economic evaluations should be mentioned in the list of eligibility criteria.

**Section: Article screening and selection**

 Will you use Ryan or Covidence to handle the study results?

 In the sentence: ‘’The checklists will be piloted on 100 random articles and screening criteria adapted accordingly’’. You could mention that you use two separate checklists for screening and selection.

 Add a sentence on the approach you will use to contact authors to obtain full-text papers if only abstracts are available.

**Section: data extraction and coding**

 Will you use NVivo V.1.7 ^10^ or QualCoder for coding?

 A draft of the data extraction sheets could be added to the protocol.

 In addition a list of example general or specific candidate items?

 What do you mean by the last part of this sentence: ‘’We will also include *specific* reporting items relevant to different categories of health economic evaluations in this and **subsequent stages of the PRISMA-EconEval project**’’? Could you please explain it?

Are the datasets presented in a usable and accessible format?

 Not applicable

Is the study design appropriate for the research question?

Yes

Is the rationale for, and objectives of, the study clearly described?

Partly

Are sufficient details of the methods provided to allow replication by others?

Partly

Are the datasets clearly presented in a useable and accessible format?

Not applicable

Reviewer Expertise:

Health Economist, Methodologist, and Epidemiologist, in the field of systematic reviews of economic evaluations, quality asssessment and reporting of economic evaluations

I confirm that I have read this submission and believe that I have an appropriate level of expertise to confirm that it is of an acceptable scientific standard, however I have significant reservations, as outlined above.
